# Multiple Query Content-Based Image Retrieval Using Relevance Feature Weight Learning

**DOI:** 10.3390/jimaging6010002

**Published:** 2020-01-17

**Authors:** Abeer Al-Mohamade, Ouiem Bchir, Mohamed Maher Ben Ismail

**Affiliations:** 1 Computer Science Department, College of Computer and Information Sciences, King Saud University, Riyadh 11362, Saudi Arabiambenismail@ksu.edu.sa (M.M.B.I.); 2 Computer Science Department, Community College, Taibah University, P.O.B.344, Madinah 41477, Saudi Arabia

**Keywords:** content-based image retrieval, multiple query, visual feature, weight learning

## Abstract

We propose a novel multiple query retrieval approach, named weight-learner, which relies on visual feature discrimination to estimate the distances between the query images and images in the database. For each query image, this discrimination consists of learning, in an unsupervised manner, the optimal relevance weight for each visual feature/descriptor. These feature relevance weights are designed to reduce the semantic gap between the extracted visual features and the user’s high-level semantics. We mathematically formulate the proposed solution through the minimization of some objective functions. This optimization aims to produce optimal feature relevance weights with respect to the user query. The proposed approach is assessed using an image collection from the Corel database.

## 1. Introduction

The proliferation of social networks along with the wide dispersion of smart devices has yielded an exponential growth of digital image databases. This massive increase has precipitated the challenge of mining specific images among huge collections. Thus, image retrieval has become an active field of research.

Text-based image retrieval systems require the annotation of images in a database. This is a tedious and expensive task. Content-based image retrieval (CBIR) represents an alternative that overcomes this drawback. Hereby, the user selects a query image that conveys the information he or she is looking for. The query content is then used to mine the database. Several CBIR approaches have been reported in the literature [1,2,3,4] during the last decade, and other CBIR applications have been proposed recently [5,6,7]. For these CBIR systems, the visual properties of an image are described using low-level feature descriptors [8]. More specifically, these descriptors can include the color, texture, and shape properties of the image. These low-level features translate the visual content of the image into numerical vectors that allow for a quantitative estimation of the similarity between two images [9,10,11,12]. In the literature, several visual descriptors have been proposed. Some of these descriptors are generic, while others are specific to some applications [13,14]. However, there is a gap between the semantic interest of the user and an extracted visual feature descriptor. For instance, if the user can provide an image containing a red apple as a query, the retrieved images may contain a red rose, red balloon, or green apple depending on the visual feature adopted by the CBIR system. Recently, multiple query retrieval systems have emerged as a way to overcome this disadvantage and to bridge the semantic gap with low-level image features [15,16]. For these systems, the user provides a set of query images. This conveys a better understanding of the user’s high-level interest to the retrieval system. The main point of multiple query CBIR systems is how to compute the distances between the set of query images and each image in the database.

Based on the investigation of existing multiple query retrieval systems, in this paper, we have designed a new approach for a multiple-query-based CBIR system, named weight-learner, which overcomes the limitations of existing approaches. The proposed approach retrieves a set of images relevant to the semantic learnt from the set of query images without learning the content of the image or its annotation. The proposed query formulation is practical for the user in such a way that 2–3 images are sufficient to express retrieval interest. Moreover, the user does not have to provide ambiguous information, such as image query scoring or feature weighting. Weight-learner expresses the semantics of the user query using feature relevance weights that are automatically learned from the set of queries. The optimal feature weights are derived through the minimization of a new formulated objective function. We should mention here that any visual feature can benefit from this approach, i.e., it is not restricted to any feature. The considered features in this work are used as examples.

In order to assess the performance of the proposed multiple query approach, we compare it to the performance of a simple query image. Moreover, we study the effects of the number of query images on weight-learner. Finally, we implement existing multiple query approaches and compare their retrieval performance to the proposed solution. The rest of the paper is organized as follows. In Section 2, we review the work related to multiple query retrieval systems. The proposed approach, weight-learner, is described in Section 3. In Section 4, we assess the performance of the weight-learner approach. Finally, in Section 5, we conclude and outline future works.

## 2. Related Works

For multiple query retrieval, the user is required to provide a set of images as query. The distance between the features of this set of images and the feature of each image in the database is then computed. The keystone of a multiple query system, then, is how to compute this distance which should reflect the information conveyed by the low-level features of the set images provided as query. In the rest of the paper, we will refer to this distance as the set distance and we will refer to the set of images that define a single query as the set of queries. In the last decade, several query retrieval systems have been proposed. In the following, we describe these approaches. 

In [17], the authors propose a weighted color and texture histogram algorithm which computes the set distance using the multi-histogram intersection method [18]. This involves a weighted combination of the texture and color distances. The color and texture weights provide the relative importance of each feature, and cross-validation technique is suggested as a way of determining these parameters. Image Grouper [19] requires the user to provide two sets of query images. One set relevant to user semantics is called multiple positive groups, and another set irrelevant to user semantics is called multiple negative groups. The set distance is computed as the distance between the mean of each positive group and the image in the database using Fisher’s discriminant analysis (FDA) [20]. The minimum weighted distance combination algorithm [21] uses a linear weighted summation of the different features. The set distance is defined as the minimum distance between each query image and the image in the dataset. Similarly, the standard-deviation-based weights approach [22] defines the set distance the same as in [21]. However, the weights are defined as the normalized inverse of the standard deviation of the image features. The MindReader approach [23] requires the user to provide a goodness score for each selected query image. Using the covariance matrix of the set of query images, this approach learns an appropriate Mahanolobis distance. The authors in [23] defined an objective function as the sum of the Mahalanobis distances of the optimal feature vector and the images in the database weighted by the corresponding goodness scores. Minimizing this objective function provides the optimal feature vector. The set distance is then expressed as the Mahanalobis distance [24] between an image in the database and the optimal feature vector. The logic AND-based distance [25] assumes that retrieved images must be similar to all query images. Thus, the set distance is expressed as the maximum of the Euclidean distances between the image query and the database images. The multi-feature query [26] combines logic AND and logic OR distances. This approach takes the minimum distance of a given query image $I_{Q}^{i}$ and a given database image $I_{D}^{j}$ with respect to the different features and the maximum of query set distances to a given image in the database. The linear distance combination approach in [27] compels the user to provide a set of query images and their respective weights or scores of goodness. The set distance is a linear weighted sum over all distances between the query images and the database image.

To the best of our knowledge, the reported approaches are the only works that deal with multiple query content based image retrieval in the literature, and although deep learning has been an active field of research, deep learning approaches have been used in the context of image retrieval only to extract the features. In fact, no deep learning-based approach has been reported to learn the sematic of the query. Concerning the approaches stated above, some require that the user provide a set of feature weights, while others compel the user to give a score of goodness set. While these weights and scores of goodness could enhance the retrieval results, they are very ambiguous to set and make the query impractical for the user. Other approaches learn the distance using the Mahanalobis distance or the standard deviation. However, these two approaches require an important number of query images to produce a significant result. This will also yield an impractical query for the user. Some other approaches use the minimum and the maximum to select a feature or a query image. This is a type of crisp weighting of the features, whereby the selected feature is assigned a value of one, and the others are assigned a value of zero. Also, when applied to query images, this approach offers a crisp score of goodness, where the selected query image is assigned a score of one. However, a selection of one feature or one query image will discard information on the remaining ones that could be useful.

## 3. Multiple Query Content-Based Image Retrieval Based on Relevance Feature Weight Learning

We propose a new approach for a multiple-query-based CBIR system, weight-learner, that overcomes the limitations of existing approaches. We make the query formulation practical for the user in such a way that 2–3 images is sufficient to express user retrieval interest. Moreover, the user does not have to provide extra information, such as image query scoring or feature weighting. We aim to express the semantics of the user query using feature relevance weights that are automatically learned from the set of query images. The weight-learner is based on a linear weighted combination of the distances between each query image and a given image in the dataset. The weight with respect to each considered feature descriptor will be learned in an unsupervised manner. These weights would allow one to discriminate between the features with respect to each query image in such a way that a large weight will be assigned to the most relevant feature. This optimization will be achieved through the minimization of the following objective function:

(1) $$J=\overset{M}{\underset{i=1}{\sum }}\overset{S}{\underset{t=1}{\sum }}w_{t}^{2}{\left(x_{\mathrm{it}}-c_{t}\right)}^{2}$$

subject to

(2) $$\forall {t\;w}_{t}\in \left[0..1\right]$$

and

(3) $$\overset{S}{\underset{t=1}{\sum }}w_{t}=1$$

where M is the number of query images, S is the number of feature entries, and $x_{\mathrm{it}}$ represents the $t^{\mathrm{th}}$ feature value of the S-dimensional data point. On the other hand, $w_{t}$ represents the discrimination weight of feature t, and $c_{t}$ is the $t^{\mathrm{th}}$ component of the center vector. The term $\sum_{t=1}^{S}w_{t}^{2}{\left(x_{\mathrm{it}}-c_{t}\right)}^{2}$ is the weighted Euclidean distance from query image $I_{Q}^{i}$ to the center of the query image set, which is defined as in Equation (4):

(4) $$c_{t}=\frac{1}{M}\overset{M}{\underset{i=1}{\sum }}x_{\mathrm{it}}$$

To optimize J with respect to $w_{t}$, we use the Lagrange multiplier technique [28] and obtain

(5) $$J^{L}=\overset{M}{\underset{i=1}{\sum }}\overset{S}{\underset{t=1}{\sum }}w_{t}^{2}{\left(x_{\mathrm{it}}-c_{t}\right)}^{2}-\lambda \left(\overset{S}{\underset{t=1}{\sum }}w_{t}-1\right).$$

The derivative with respect to $w_{t}$ is

(6) $$\frac{\partial J^{L}}{\partial w_{t}}=2w_{t}\overset{M}{\underset{i=1}{\sum }}{\left(x_{\mathrm{it}}-c_{t}\right)}^{2}-\lambda .$$

Setting the derivative to zero yields

(7) $$w_{t}=\frac{\lambda }{2\sum_{i=1}^{M}{\left(x_{\mathrm{it}}-c_{t}\right)}^{2}}.$$

The derivative with respect to λ is

(8) $$\frac{\partial J^{L}}{\partial \lambda }=\overset{S}{\underset{t=1}{\sum }}w_{t}-1.$$

Setting $\frac{\partial J^{L}}{\partial \lambda }$ to 0 gives

(9) $$\overset{S}{\underset{t=1}{\sum }}w_{t}=1.$$

Substituting Equation (7) in (9) gives

(10) $$\overset{S}{\underset{t=1}{\sum }}\frac{\lambda }{2\sum_{i=1}^{M}{\left(x_{it}-c_{t}\right)}^{2}}=1$$

which yields

(11) $$\lambda =\frac{2}{\sum_{t=1}^{S}\frac{1}{\sum_{i=1}^{M}{\left(x_{it}-c_{t}\right)}^{2}}}$$

from which,

(12) $$w_{t}=\frac{\frac{1}{\sum_{i=1}^{M}{\left(x_{\mathrm{it}}-c_{t}\right)}^{2}}}{\sum_{q=1}^{S}\frac{1}{\sum_{i=1}^{M}{\left(x_{\mathrm{iq}}-c_{q}\right)}^{2}}}.$$

Simplifying Equation (13), we obtain

(13) $$w_{t}=\frac{D_{t}}{\sum_{q=1}^{S}D_{q}}$$

where $D_{t}=1/\sum_{i=1}^{M}{\left(x_{\mathrm{it}}-c_{t}\right)}^{2}$, which reflects the inverse of the dispersion of the set of query images along the $t^{\mathrm{th}}$ dimension, and $\sum_{q=1}^{S}D_{q}$ can be perceived as the overall inverse dispersion of the set of query images. Therefore, $w_{t}$ is inversely proportional to the ratio of the dispersion along the $t^{\mathrm{th}}$ dimension to the total image query dispersion. Thus, the more compact the set of queries is along the $t^{\mathrm{th}}$ dimension, the higher the discriminating weight, $w_{t}$, will be for the $t^{\mathrm{th}}$ dimension. Once the set of weights, $w_{t}$, is computed using Equations (4) and (12), the distance between the set of query images, $I_{Q}={\left[x_{\mathrm{it}}\right]}_{\begin{array}{c} 1\leq i\leq M\\ 1\leq t\leq S \end{array}}$, and the images in the dataset, $I_{D}^{j}={\left[x_{\mathrm{jt}}\right]}_{\begin{array}{c} 1\leq j\leq N\\ 1\leq t\leq S \end{array}}$, is defined as

(14) $$dist\left(I_{Q},I_{D}^{j}\right)=\overset{M}{\underset{i=1}{\sum }}\;\overset{S}{\underset{t=1}{\sum }}w_{t}^{2}{\left(x_{\mathrm{it}}-x_{\mathrm{jt}}\right)}^{2}.$$

Algorithm 1 describes the steps of the proposed approach.
**Algorithm 1** Weight-learnerInput: ■$I_{Q}=\left\{I_{Q}^{i}\left(i=1,\ldots ,M\right)\right\}$: set of M query images,■$I_{D}=\left\{I_{D}^{j}\left(j=1,\ldots ,N\right)\right\}$: image dataset of size N
Output: K retrieved images
Extract the set of feature descriptors from each image $I_{D}=\left\{I_{D}^{j}\left(j=1,\ldots ,N\right)\right\}$Extract the set of feature descriptors from each query $I_{Q}=\left\{I_{Q}^{i}\left(i=1,\ldots ,M\right)\right\}$Compute the weights using (4) and (12),For each image $I_{D}^{j}$ in the database (j=1,…,N)  Compute the distance $D\left(I_{Q}^{1},\ldots ,I_{Q}^{M},I_{D}^{j}\right)$ between the set of query images $I_{Q}$ and an image $I_{D}^{j}$ using (14)Sort $D\left(I_{Q}^{1},\ldots ,I_{Q}^{M},I_{D}^{j}\right)$, (j=1,…,N) in increasing orderReturn the K first images with the smallest distances

## 4. Experiments

In this chapter, we assess the performance of the proposed weight-learner approach using image collection from the Corel dataset [29]. In the first experiment, we compare the performance of the proposed multiple query approach to the performance of a simple query image. In the second experiment, we study the effect of the number of query images on the proposed weight-learner approach. In the third experiment, we implement existing multiple query approaches and compare their retrieval performances to the proposed solution. In the following section, a description of the considered dataset, an outline of the retrieval performance measure used, and details about the performed experiments are provided.

### 4.1. Dataset Description

In order to assess the performance of the proposed approach, we use a subset of 1300 images from the Corel dataset [29]. This dataset includes 12 categories: air shows, arabian horse, antelope, beach, bears, birds, butterfly, bulling, buses, car performance, car race, and dogs. Each class contains 100 images, except for the last category, which contains 200 images.

### 4.2. Experimental Setting

Since any visual feature can benefit from this approach and it not restricted to any feature, from the 1300 images, we extract the color histogram [30] and the edge histogram features [31] as examples. After concatenating the two features, entries between 1 and 150 correspond to the edge histogram, and entries between 151 and 341 correspond to the color histogram. We set this number of query images to five, as we consider more than five query images to be impractical for the user. Next, we build 260 groups of five images randomly selected from each category to be used as query sets for every analysis.

For multiple query approaches that require feature weights, we considered three cases. Table 1 summarizes the three considered cases for the two feature weights, WC and WT.

For the other multiple query approaches requiring a goodness score of v, we attribute a score of 1 to 5 to the five query images. We consider five cases obtained by permuting the scores between the query images. Table 2 reports the five considered cases.

We use the average normalized modified rank retrieval (ANMRR) score [32] to assess the performance of the multiple query approaches. The ANMRR score is between zero and one. This value is small for good retrieval results and large for bad retrieval results.

### 4.3. Experiment 1

In order to show that the weight-learner reduces the semantic gap between the extracted visual features and the user’s high-level semantics, we will compare the results of the proposed approach to a classical single query retrieval result. For each of the two systems, the proposed approach and the single query image, we will convey the edge histogram feature, the color histogram feature, and the concatenation of both. Table 3 reports a comparison of the performance of the proposed multiple query approach vs. the performance of a single query image using an edge histogram, color histogram, and the concatenated features based on ANMRR. For the three considered cases, the proposed multiple query approach gives a lower ANMRR score. This means that weight-learner outperforms the single query approach. Moreover, we notice from Table 3 that, in the case of a single query approach, the performance decreases when using both features. This decrease in performance is due to the problem of dimensionality. This effect will be worse if more features are used. Since the feature descriptor choice for a specific query is challenging, a consideration of all features will lower the performance of the system. However, the proposed multiple query approach has a low ANMRR (0.2906) when using both features, reflecting a very good retrieval result. Indeed, weight-learner is based on a linear weighted combination of the distances between each query image and a given image in the dataset. These weights allow one to discriminate between the features with respect to each query image in such a way that a large weight will be assigned to the most relevant feature. These feature relevance weights that are learned from the set of query images express the semantics of the user query.

**Figure 1:**
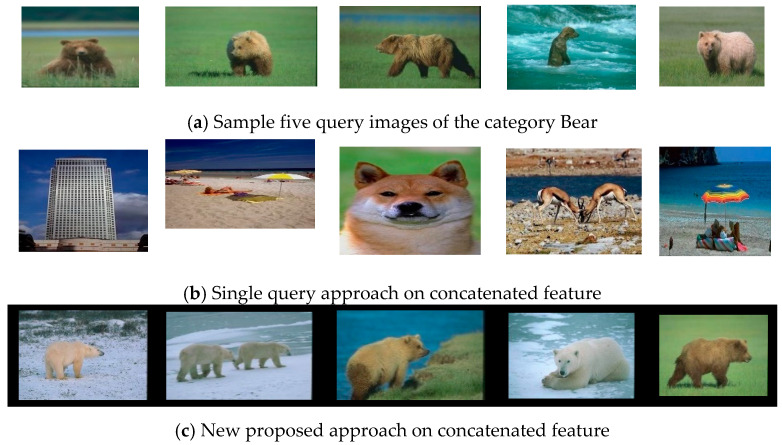
Retrieval results of the sample query for the category “bears” using concatenated features. (**a**) The 5 query images, (**b**) the corresponding retrieval result using a single query approach (only the first query image is used), and (**c**) the corresponding retrieval result using the proposed approach.

In order to show examples of how the proposed approach outperforms the single query approach, we display in Figure 1 the retrieval results of the sample query for the category Bears using concatenated features. Figure 1a shows the five query images provided as input for the proposed approach. Figure 1b reports the retrieval result using a single query approach, where only the first query image is used. Figure 1c displays the retrieval results using the new proposed approach. The single query approach does not return any correct images among the first retrieved images. However, the five retrieved images using weight-learner are all correct.

### 4.4. Experiment 2

**Figure 2:**
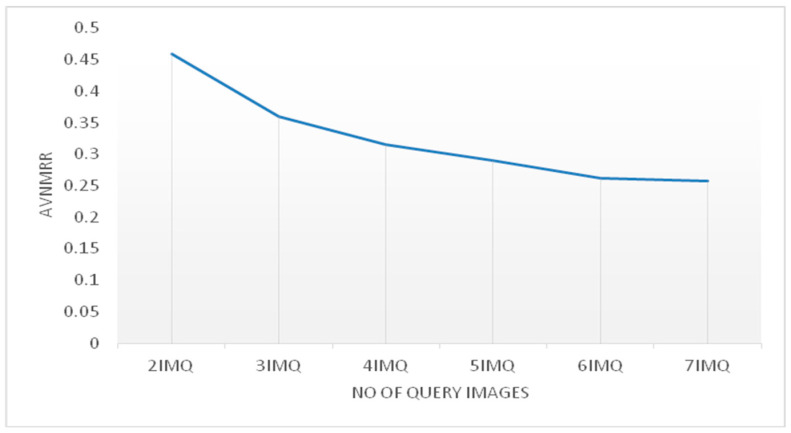
Proposed approach performance with respect to the number of query images.

In this experiment, we aim to study the effect of the number of query images on the proposed approach. We will convey both features to the proposed multiple query approach and tune the number of query images from two to seven and record the ANMRR score for each considered image query number. Figure 2 reports the proposed approach performance with respect to the number of query images. Ultimately, the AVNMRR decreases when the number of query images increases. This is expected since more query images would provide more information and thus guide the proposed retrieval system towards a better retrieval result. Moreover, the decrease rate becomes slower starting at four query images. Table 4 shows the performance of the proposed multiple query approach when the number of query images is equal to two compared to the performance of a simple query image using the concatenated features based on ANMRR. Even when using only two query images, the performance of the retrieval system increases drastically. We can thus conclude that the proposed approach has a query formulation that is practical for the user in such a way that 2–3 images would be sufficient to express retrieval interest.

### 4.5. Experiment 3

In this experiment, we compare the proposed multiple query image to the existing multiple query images described in Section 2. Namely, we compare the proposed approach to the weighted color and texture histograms approach [17], the Image Grouper approach [19], the Minimum weighted distance combination approach [21], the standard-deviation-based weights approach [22], the MindReader approach [23], the Logic AND-based distance approach [25], the Multi-feature query approach [26], and the distance combination [27] approach. In this experiment, we consider five query images for all approaches since we assume that five query images are the maximum number of query images that can be considered for the query to remain practical for the user.

Table 5 compares the performance of the proposed multiple query approach to the existing multiple query approaches and to the single query approach based on ANMRR. The proposed approach noticeably outperforms all existing approaches. Indeed, our approach has the lowest ANMRR when using the concatenated feature (0.2906). Moreover, even when using color features or textures feature only, the proposed approach still outperforms the other approaches. Moreover, the weighted color and texture histograms approach [17] has a lower performance than the single query approach even when considering the best results over the five considered feature weight scenarios. This performance discrepancy is due to the sensitivity to the choice of the feature weights. The Multi-feature query approach [26] with an ANMRR of 0.4266 is the next best multiple query retrieval system after the proposed one. Although this approach does not formally use feature weights (refer to Section 2), by taking the minimum over the feature values, it offers a type of crisp weighting, where the feature with the minimum value is assigned a value of 1, and all the others have a value of 0. This explains why this model outperforms the other existing approaches. However, this other approach takes into account only one feature and discards the others, so the information provided by the other features is lost. Since our approach uses a weighted combination of all features, all the information provided by the features is taken into account with different levels of importance according to the weight value. These weights allow for discrimination between the different feature values. This explains the outstanding performance of the proposed approach. Moreover, the proposed approach does not require any feature weight tuning since the feature weights are automatically learnt from the user’s set of queries.

**Figure 3:**
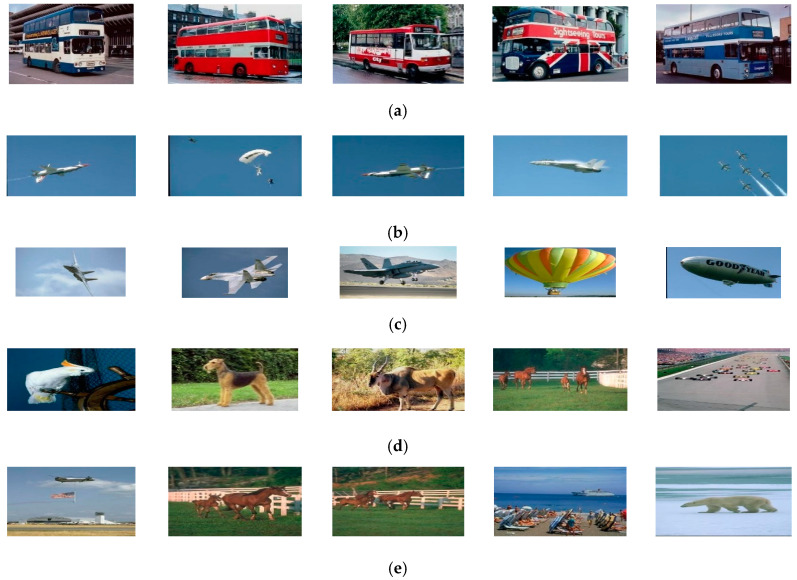

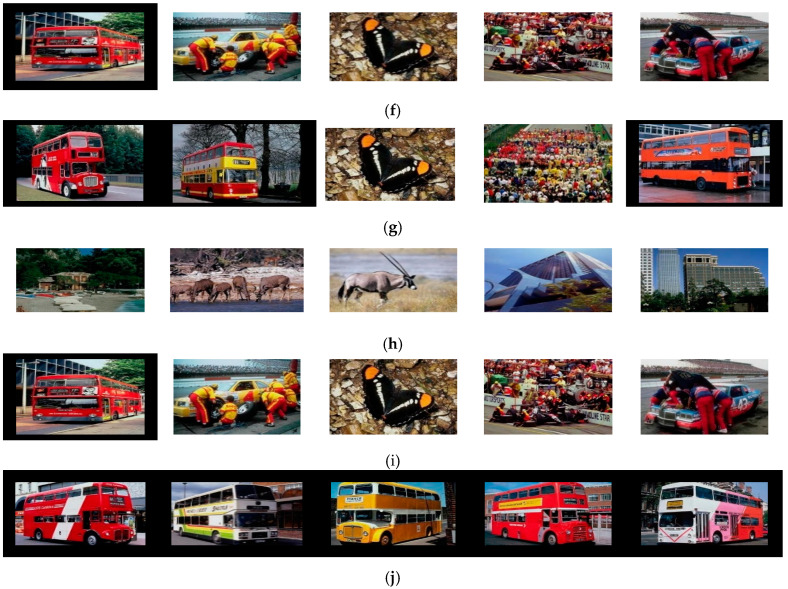
Sample query 2 and its corresponding retrieval result with respect to all considered approaches. (**a**) The query images. (**b**) Results obtained using the method in [17] with color weight = 0.1 and texture weight = 0.9. (**c**) Results obtained using the Image Grouper [19]. (**d**) Results obtained using the method in [22] with color weight = 0.5 and texture weight = 0.5. (**e**) Results obtained using the method in [22]. (**f**) Results obtained using the method in [23]. (**g**) Results obtained using the method in [26]. (**h**) Results obtained using the method in [27]. (**i**) Results obtained using the method in [29]. (**j**) Results obtained using the proposed method.

To further investigate how the proposed approach functions better than the other approaches, we provide a sample query set corresponding to the category of buses (Figure 3). Figure 3a shows the corresponding five query images. Figure 3b–f,i reports the first five retrieved images returned by the existing approaches. For each existing approach, the parameters giving the best results are considered. The last set of images (Figure 3j) is related to the proposed approach. The images highlighted by the black box show that the image was correctly retrieved.

**Figure 4:**
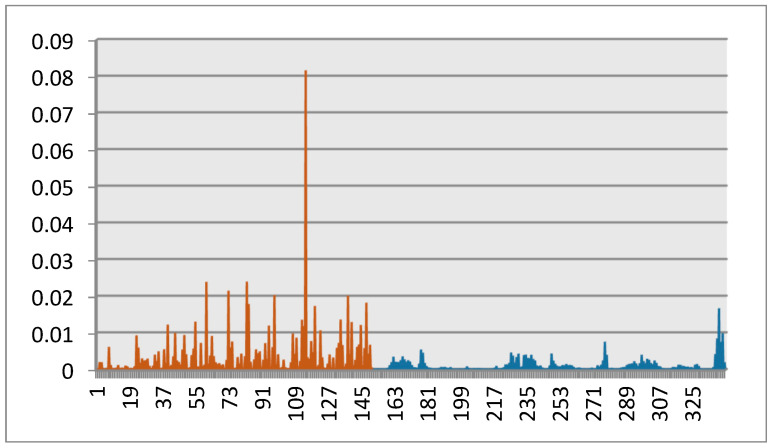
Learned weights using the new proposed approach on Sample query 2. Entries between 1 and 150 correspond to the edge histogram, and entries between 151 and 341 correspond to the color histogram.

From Figure 3, we can see that, for some existing multiple query approaches, the “bus” category does not appear in the set of retrieved images while images related to other categories are retrieved. This means that these approaches failed to understand the semantic “bus” of the query images. On the other hand, our approach noticeably outperforms the other approaches. This means that the proposed approach is able to successfully capture user semantics through the conveyed set of query images. This is done by learning the appropriate feature relevance weights that discriminate between features. Figure 4 plots the weights learned by the proposed approach for the considered sample query set. Entries between 1 and 150 correspond to the edge histogram, and entries between 151 and 341 correspond to the color histogram. We can see from Figure 4 that the edge histogram entries are assigned automatically larger weights than the color histogram ones. This means that the various colors of the buses are not taken into account, but the shapes of the buses are considered. This yields a successful retrieval result. The proposed approach looks at the similarities between the different feature entries of the same query set and learns the appropriate relevance feature weights to yield outperforming retrieval results.

## 5. Conclusions

Despite the availability of advanced visual image low-level descriptors, understanding the semantic concept of an image remains a challenging task. It is even harder to express the interest of the user using one single image. Multiple query images help to better express user interest and narrow the gap between an image’s semantics and its visual descriptors. However, a multiple-query-based CBIR system should be practical and able to effectively aggregate the information provided by different query images to enhance retrieval performance.

In this work, we proposed a novel linear weighted combination of distances between each query image and the images in the database. We learned the relevance weight for each query image with respect to each visual descriptor entry. These weights allowed us to discriminate between the features in such a way that large weights are assigned to the most relevant feature entry. These weights are learned through the minimization of an appropriate objective function. The experimental results show that weight-learner outperforms single query retrieval results. Moreover, it makes query formulation practical for the user in such a way that two to three images would be sufficient to express a retrieval interest.

For future work, we intend to investigate the image query goodness score. We plan to learn a weight *w_it_* for each image query *i* with respect to the feature entry *t*. This way, the learned weights will reflect both the relevance feature weight and the image query goodness score. This work will yield a new definition for the distance between a set of query images and the images of the database. We also intend to use the new distance of the proposed approach as a way of including the feedback of the user in the retrieval. Thus, instead of considering a set of multiple query images, we use a set of feedback images reported as correctly retrieved by the user. The learned weight would then improve the next query.

## Figures and Tables

**Table 1:** The three considered cases for manually set feature weights.

Considered Cases	WC	WT
First Case	0.1	0.9
Second Case	0.5	0.5
Third Case	0.9	0.1

**Table 2:** The five considered cases for manually set query image scores.

Considered Cases	v1	v2	v3	v4	v5
First Case	1	2	3	4	5
Second Case	2	3	4	5	1
Third Case	3	4	5	1	2
Fourth Case	4	5	1	2	3
Fifth Case	5	1	2	3	4

**Table 3:** Comparison of the performance of the proposed multiple query approach vs. the performance of a simple query image using a texture histogram, color histogram, and concatenated features based on average normalized modified rank retrieval (ANMRR).

Approaches	Texture Histogram	Color Histogram	Concatenated Features
Simple Query Image	0.6852	0.692	0.7058
Proposed Multiple Query	0.3959	0.301	0.2906

**Table 4:** The performance of the proposed multiple query approach when the number of query images is equal to 2 compared to the performance of a simple query image using the concatenated features based on ANMRR.

Approaches	ANMRR
Single Query Image	0.7058
Proposed Multiple Query Approach When the Number of Query Images is Equal to 2	0.459512

**Table 5:** The performance of the proposed multiple query approach compared to the existing multiple query approaches and to the single query approach based on ANMRR.

Name of Multiple Query System	ANMRR
The weighted color and texture histograms approach [17]	0.8616
The Image Grouper approach [19]	0.693
The Minimum weighted distance combination approach [21]	0.5096
The standard-deviation-based weights approach [22]	0.5251
The MindReader approach [23] (D5)	0.4522
The Logic AND-based distance approach [26]	0.4536
The Multi-feature query approach [27]	0.4266
The distance combination [29] approach	0.4522
Single query based on color histogram	0.6852
Single query based on texture histogram	0.7058
Single query based on concatenated feature	0.692
New proposed approach based on color histogram	0.301
New proposed approach based on texture histogram	0.3959
New proposed approach based on concatenated feature	0.2906
